# Ectopic insert-dependent neuronal expression of GFAP promoter-driven AAV constructs in adult mouse retina

**DOI:** 10.3389/fcell.2022.914386

**Published:** 2022-09-19

**Authors:** Nguyet Le, Haley Appel, Nicole Pannullo, Thanh Hoang, Seth Blackshaw

**Affiliations:** ^1^ Solomon H. Snyder Department of Neuroscience, Johns Hopkins University School of Medicine, Baltimore, MD, United States; ^2^ Department of Neurology, Johns Hopkins University School of Medicine, Baltimore, MD, United States; ^3^ Department of Ophthalmology, Johns Hopkins University School of Medicine, Baltimore, MD, United States; ^4^ Institute for Cell Engineering, Johns Hopkins University School of Medicine, Baltimore, MD, United States; ^5^ Kavli Neuroscience Discovery Institute, Johns Hopkins University School of Medicine, Baltimore, MD, United States

**Keywords:** glia, reprogramming, muller glia, adeno associated viral vectors, adeno-associated virus, glial fibrillary acidic protein, ectopic, cell lineage analysis

## Abstract

Direct reprogramming of retinal Müller glia is a promising avenue for replacing photoreceptors and retinal ganglion cells lost to retinal dystrophies. However, questions have recently been raised about the accuracy of studies claiming efficient glia-to-neuron reprogramming in retina that were conducted using GFAP mini promoter-driven adeno-associated virus (AAV) vectors. In this study, we have addressed these questions using GFAP mini promoter-driven AAV constructs to simultaneously overexpress the mCherry reporter and candidate transcription factors predicted to induce glia-to-neuron conversion, in combination with prospective genetic labeling of retinal Müller glia using inducible Cre-dependent GFP reporters. We find that, while control GFAP-mCherry constructs express faithfully in Müller glia, 5 out of 7 transcription factor overexpression constructs tested are predominantly expressed in amacrine and retinal ganglion cells. These findings demonstrate strong insert-dependent effects on AAV-based GFAP mini promoter specificity that preclude its use in inferring cell lineage relationships when studying glia-to-neuron conversion in retina.

## Introduction

Retinal degeneration is characterized by irreversible loss of retinal neurons and is a major cause of blindness. Macular degeneration and retinitis pigmentosa induce rod and cone photoreceptor loss, while glaucoma leads to retinal ganglion cell degeneration. Two main classes of cell-based therapies are being actively pursued to replace these lost neurons. The first of these involves direct differentiation and transplantation of embryonic stem (ES)/induced pluripotent stem (iPS)-derived cells, for which large numbers of the desired cell type can be produced on demand (Osakada et al., 2008; Lamba et al., 2010; Tucker et al., 2013). However, this approach has thus far had limited success, largely because of the limited efficiency of integration of transplanted cells into host circuitry, particularly in diseased retina (Gasparini et al., 2019; Miltner and La Torre, 2019; Singh et al., 2020; Zhang and Wang, 2022). The second approach takes inspiration from injury-induced regeneration that occurs in many cold-blooded vertebrates, and involves directed reprogramming of retinal Müller cells into neurogenic progenitors and/or retinal neurons (Goldman, 2014; Lahne et al., 2020; Salman et al., 2021). Glial reprogramming has a number of potential therapeutic advantages relative to exogenous cell transplantation, including the reduced likelihood of immune rejection and the fact that glial-derived neurons are already present in the retina itself.

Studies using transgenic mice for cell lineage tracing have shown that either overexpression of the neurogenic bHLH factor *Ascl1* (Jorstad et al., 2017; Todd et al., 2020; Palazzo et al., 2022), or genetic disruption of NFI family transcription factors (Hoang et al., 2020) induces Müller glia to generate bipolar and amacrine interneurons, but neither photoreceptors nor mature retinal ganglion cells, although a recent report has shown that simultaneous overexpression of *Atoh1* and *Ascl1* can induce formation of immature retinal ganglion cells (Todd et al., 2021). In contrast, several studies using adeno-associated virus (AAV)-mediated transcription factor overexpression (Yao et al., 2018; Xiao et al., 2021) or CasRx-mediated knockdown of the splicing regulator *Ptbp1* (Fu et al., 2020; Zhou et al., 2020) have reported highly efficient generation of photoreceptor and retinal ganglion cells from Müller glia.

Particularly in the light of similar studies reporting efficient astrocyte-to-neuron reprogramming in brain (Brulet et al., 2017; Qian et al., 2020; Zhou et al., 2020; Xiang et al., 2021), AAV-based glia-to-neuron conversion has been hailed as a broadly promising approach for treatment of many classes of neurodegenerative disorders (Janowska et al., 2019; Wang Y. et al., 2021; Götz and Bocchi, 2021). However, several important technical concerns have arisen about many of these studies. First, while studies reporting Müller glia-to-neuron conversion using transgenic mice have used well-characterized inducible transgenic Cre lines to infer cell lineage relationships (Jorstad et al., 2017; Hoang et al., 2020), AAV-based studies have used GFAP minipromoter-driven Cre constructs for this purpose. Second, the GFAP minipromoter, which is used to drive glial-specific expression of constructs used for reprogramming, can show leaky neuronal expression in brain (Taschenberger et al., 2017; Griffin et al., 2019), most notably when overexpressing *Neurod1* or disrupting *Ptbp1* expression in brain astrocytes (Wang L.-L. et al., 2021; Chen et al., 2021), both of which have been claimed to induce astrocyte-to-neuron conversion (Puls et al., 2020; Qian et al., 2020; Zhou et al., 2020; Xiang et al., 2021). This raises the question of whether reports of AAV-based Müller glia-to-neuron conversion actually represent cases of ectopic expression of GFAP reporter constructs in endogenous neurons (Blackshaw and Sanes, 2021; Qian et al., 2021).

To directly address this question, we used tamoxifen treatment to selectively label Müller glia in adult mouse retina using the well-characterized *GlastCreERT2;Sun1-GFP* transgenic line (de Melo et al., 2012, 2016; Hoang et al., 2020). We then performed intravitreal injection in these retinas with GFAP promoter-driven 7m8 AAV2 constructs expressing either mCherry alone or mCherry in combination with a range of different transcription factors predicted to potentially induce glia-to-neuron conversion. We found that Sun1-GFP expression was restricted to Müller glia, but that a number of constructs overexpressing transcription factors showed leaky mCherry expression in multiple inner retinal neuronal subtypes, including retinal ganglion cells. This demonstrates widespread insert-dependent induction of leaky neuronal expression of GFAP minipromoter-driven constructs and underlines the importance of using well-characterized genetic cell lineage analysis when studying glia-to-neuron conversion.

## Results

**FIGURE 1:**
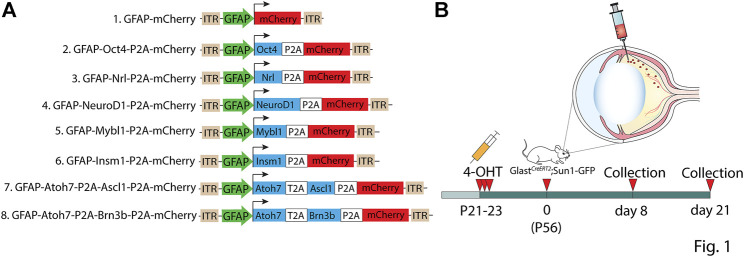
Summary of constructs tested in the study. **(A)** Schematic of the GFAP AAV constructs and **(B)** the workflow: three weeks old *GlastCreERT2;Sun1-GFP* mice were induced with 3 daily doses of 4-OHT delivered intraperitoneally, intravitreally injected with GFAP AAV constructs at 2 months of age, and collected 8 days and 21 days following AAV infection for analysis.

We sought to measure Müller glia-to-neuron conversion following overexpressing of candidate transcription factors using GFAP minipromoter-based 7m8 AAV2 vectors in the adult mouse retina, which have been previously shown to selectively restrict expression of fluorescent proteins to Müller glia in uninjured adult retina (Aartsen et al., 2010; Hickey et al., 2017; Yao et al., 2018; Zhou et al., 2020). Constructs tested included a GFAP-mCherry control and a range of different transcription factors that have been previously reported to reprogram Müller glia or were candidates for doing so based on their role in regulating neurogenesis and/or retinal neuron specification (Figure 1A) (Mears et al., 2001; Sharma et al., 2019; Puls et al., 2020; Lyu et al., 2021; Todd et al., 2021; Xiao et al., 2021). To genetically label Müller glia prior to AAV infection, we conducted 3 consecutive intraperitoneal injections of 4-hydroxytamoxifen (4-OHT) from P21-P23 in *GlastCreERT2;Sun1-GFP* mice (Figure 1B). At 2 months of age–roughly the age used for previous studies reporting Müller glia-to-neuron conversion–we then performed intravitreal AAV injection, and analyzed retinas at 8 and 21 days later (Figure 1B). *GlastCreERT2;Sun1-GFP* mice show selective and robust perinuclear GFP labeling which colocalizes with selective Müller glial marker Sox9, although a few Sox9/GFP-positive astrocyte nuclei are observed in the ganglion cell layer (Supplementary Figures S1A,B). No additional injury was administered to the retina, as all previous studies that reported direct and efficient GFAP AAV-dependent Müller glial-to-neuron conversion observed this in uninjured retinas (Yao et al., 2018; Fu et al., 2020; Zhou et al., 2020; Xiao et al., 2021).

**FIGURE 2:**
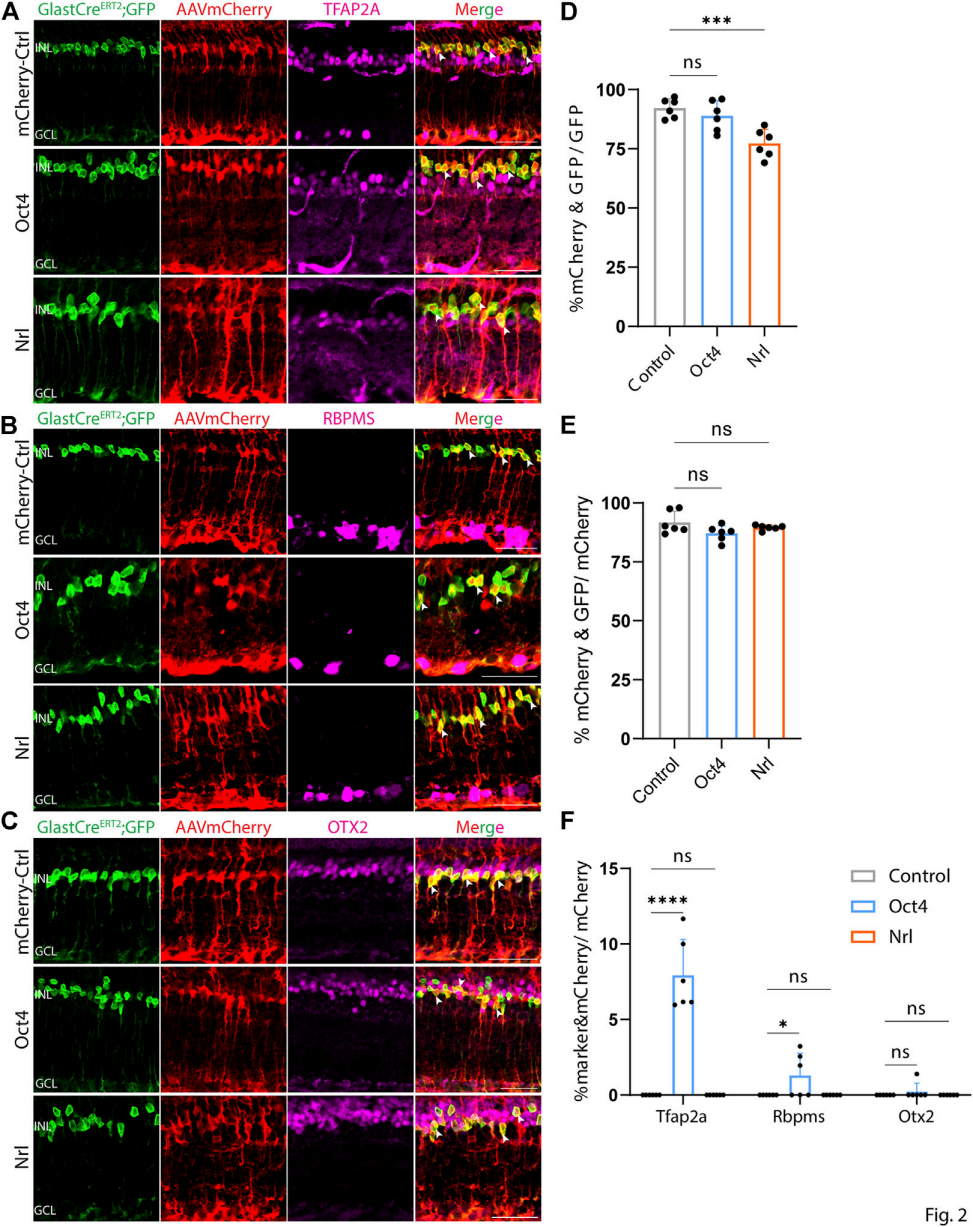
GFAP promoter constructs expressing mCherry, Nrl, and Oct4 show strong Müller glia-specific expression. Representative immunostaining for GFP, mCherry and 3 neuronal markers: **(A)** Tfap2a, **(B)** Rbpms, and **(C)** Otx2 expression in the retinas collected 21 days post GFAP AAV infection. GFAP-mCherry, GFAP-Oct4-mCherry and GFAP-Nrl-mCherry showed almost complete colocalization of construct-derived mCherry and Müller glia-specific Sun1-GFP. White arrowheads indicate co-labeled mCherry+/GFP + cells. Little to no mCherry expression in amacrine, retinal ganglion and bipolar cells is observed in these GFAP AAV constructs. Quantification of transduction efficiency (mCherry+ & GFP + cells/GFP + cells) **(D)** and transduction specificity (mCherry+ & GFP + cells/mCherry + cells) **(E)**. Quantification of mean percentage ±SD of marker & mCherry+/GFP cells: Tfap2a+ & mCherry+/GFP + cells (in INL and GCL), Rbpms+ & mCherry+/GFP + cells (in GCL) and Otx2+&mCherry+/GFP + cells (in INL) **(F)**. Significance was determined *via* one-way ANOVA or two-way ANOVA with Dunnett’s test: ***p < 0.001, ****p < 0.0001. Each data point in the bar graphs was calculated from an individual retina (*n* = 6). INL, inner nuclear layer; GCL, ganglion cell layer. Scale bar = 40 μm.

**FIGURE 3:**
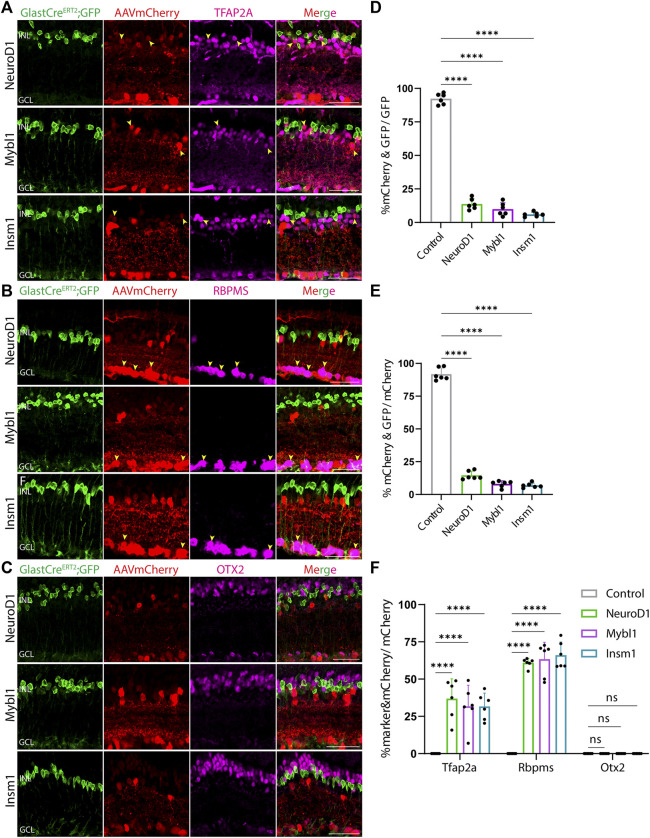
GFAP promoter constructs expressing NeuroD1, Mybl1 and Insm1 show ectopic mCherry expression in amacrine and ganglion cells. Representative immunostaining for GFP, mCherry and 3 neuronal markers: **(A)** Tfap2a, **(B)** Rbpms, and **(C)** Otx2 expression in the retinas collected 21 days post GFAP AAV infection. GFAP-NeuroD1-mCherry, GFAP-Mybl1-mCherry and GFAP-Insm1-mCherry showed little to no colocalization of construct-derived mCherry and Müller glia-specific Sun1-GFP. High levels of mCherry expression in amacrine and ganglion cells, but not in bipolar cells were observed in retinas infected with these GFAP AAV constructs. Yellow arrowheads indicate co-labeled mCherry+ & marker + cells. Quantification of transduction efficiency (mCherry+ & GFP + cells/GFP + cells) **(D)** and transduction specificity (mCherry+ & GFP + cells/mCherry + cells) **(E)**. Quantification of mean percentage ±SD of marker & mCherry+/GFP cells: Tfap2a+ & mCherry+/GFP + cells (in INL and GCL), Rbpms+ & mCherry+/GFP + cells (in GCL) and Otx2+&mCherry+/GFP + cells (in INL) **(F)**. Significance was determined *via* one-way ANOVA or two-way ANOVA with Dunnett’s test: ****p < 0.0001. Each data point in the bar graphs was calculated from an individual retina (*n* = 6). INL, inner nuclear layer; GCL, ganglion cell layer. Scale bar = 40 μm.

**FIGURE 4:**
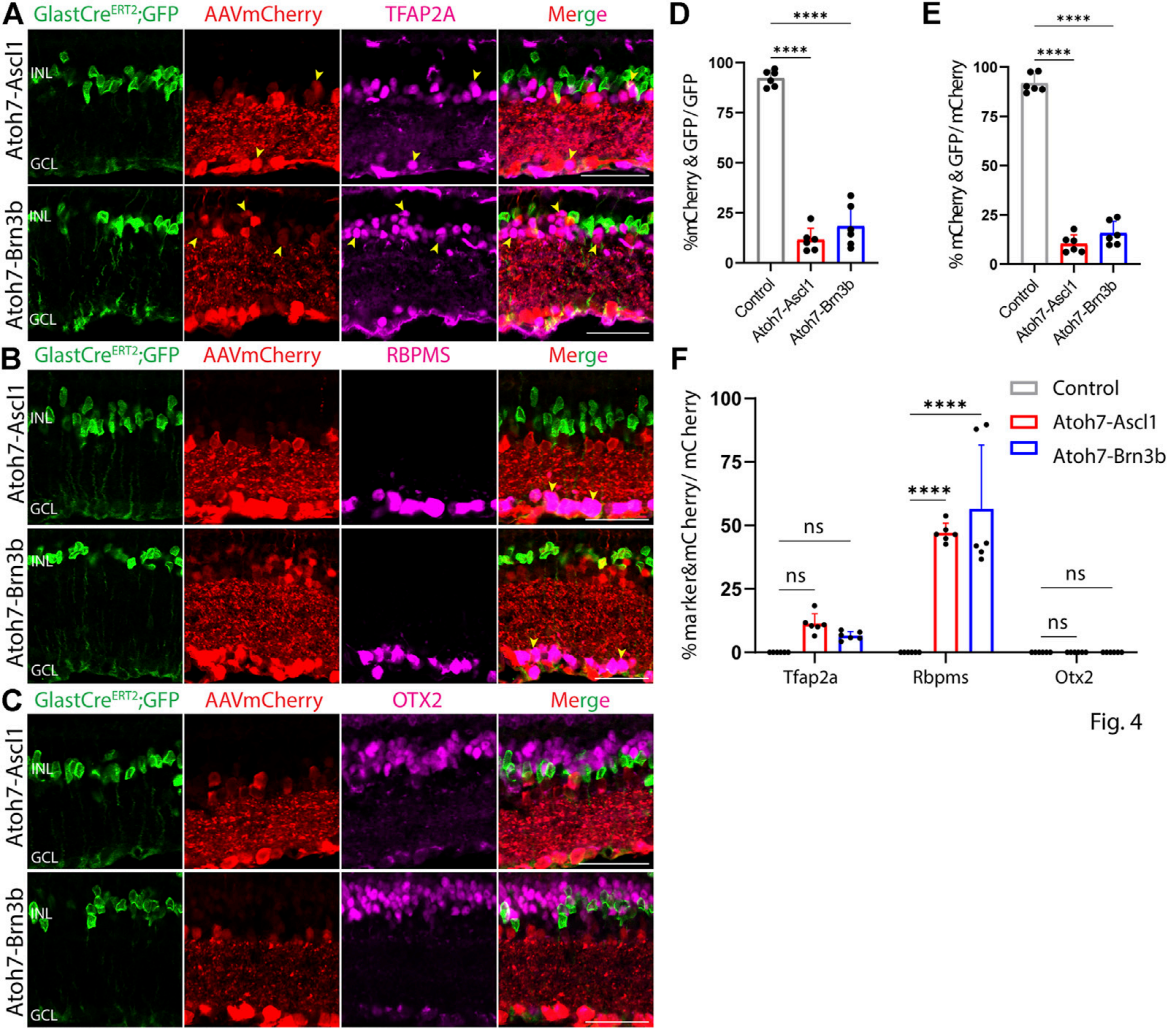
GFAP promoter constructs tandemly expressing Atoh7-Ascl1 and Atoh7-Brn3b show ectopic mCherry expression in amacrine and ganglion cells. Representative immunostaining for GFP, mCherry and 3 neuronal markers: **(A)** Tfap2a, **(B)** Rbpms, and **(C)** Otx2 expression in the retinas collected 21 days post GFAP AAV infection. GFAP-Atoh7-Ascl1-mCherry and GFAP-Atoh7-Brn3b-mCherry showed little to no colocalization of construct-derived mCherry and Müller glia-specific Sun1-GFP. High levels of mCherry expression in amacrine and ganglion cells, but not in bipolar cells were observed in retinas infected with these GFAP AAV constructs. Yellow arrowheads indicate co-labeled mCherry+ & marker + cells. Quantification of transduction efficiency (mCherry+ & GFP + cells/GFP + cells) **(D)** and transduction specificity (mCherry+ & GFP + cells/mCherry + cells) **(E)**. Quantification of mean percentage ±SD of marker & mCherry+/GFP cells: Tfap2a+ & mCherry+/GFP + cells (in INL and GCL), Rbpms+ & mCherry+/GFP + cells (in GCL) and Otx2+&mCherry+/GFP + cells (in INL) **(F)**. Significance was determined *via* one-way ANOVA or two-way ANOVA with Dunnett’s test: ****p < 0.0001. Each data point in the bar graphs was calculated from an individual retina (*n* = 6). INL, inner nuclear layer; GCL, ganglion cell layer. Scale bar = 40 μm.

At both 8 and 21 days after AAV injection, we saw considerable differences in the pattern of cell type-specific expression of mCherry among the different constructs tested. GFAP-mCherry control, GFAP-Oct4-mCherry, and GFAP-Nrl-mCherry all showed almost complete colocalization of mCherry expression with Müller glia-specific Sun1-GFP (Figures 2A–C). Following Oct4 overexpression, a small number of mCherry-expressing cells also expressed the amacrine cell-specific marker Tfap2a (Figure 2A) or the ganglion cell-specific marker Rbpms (Figure 2B), while no mCherry-expressing cells were observed following overexpression of Nrl. Neither Oct4 nor Nrl overexpressing resulted in mCherry expression in any cells expressing the bipolar/photoreceptor cell-specific marker OTX2 (Figure 2C) (Lyu et al., 2021). Immunostaining for Nrl showed colocalization with mCherry-positive cells in GFAP-Nrl-mCherry-transduced cells (Supplementary Figure S5). All other constructs tested–including GFAP-Neurod1-mCherry, GFAP-Mybl1-mCherry, GFAP-Insm1-mCherry, GFAP-Atoh7-Ascl1-mCherry, and GFAP-Atoh7-Brn3b-mCherry–showed little mCherry expression in Sun1-GFP-positive Müller glia (Figures 3A–E, Figures 4A–E). Instead, robust mCherry expression was detected in the inner retinal neurons, specifically retinal amacrine cells (Figures 3A,F) and ganglion cells (Figures 3B,F), although no mCherry expression was seen in Otx2-positive retinal bipolar cells for any of the constructs tested (Figures 3C,F). This pattern of retinal neuronal expression was also evident at 8 days following infection for GFAP-Insm1-mCherry (Supplementary Figure S2), GFAP-Atoh7-Ascl1-mCherry, and GFAP-Atoh7-Brn3b-mCherry (Supplementary Figure S3).

**FIGURE 5:**
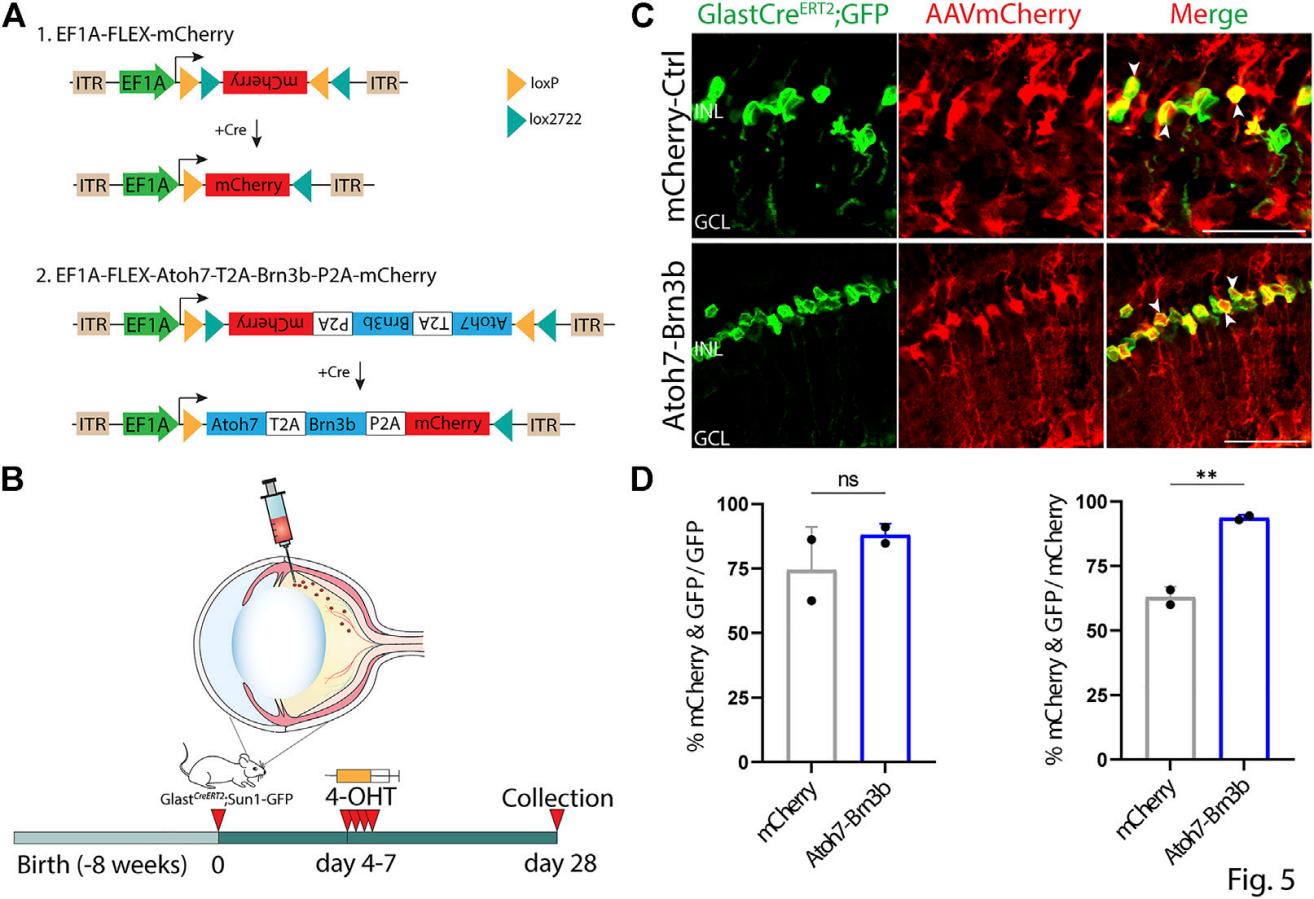
Inserts that show ectopic neuronal expression when driven by the GFAP promoter are restricted to Müller glia when expressed using GlastCreERT2-dependent EF1a FLEX AAV constructs. **(A)** A schematic of Cre-dependent FLEX-mCherry control and FLEX-Atoh7-Brn3b-mCherry constructs. **(B)** A schematic of the experimental workflow: two months old *GlastCreERT2;Sun1-GFP* mice were intravitreally injected with FLEX AAV constructs and collected 21 days following the 4th dose of 4-OHT i.p injection for analysis. **(C)** Representative immunostaining for GFP and mCherry expression in FLEX AAV infected retinas collected 21 days post Cre-recombination induction. FLEX-Atoh7-Brn3b-mCherry showed extensive colocalization of GFP and mCherry. White arrowheads indicate mCherry+/GFP + cells. **(D)** Quantification of mean percentage ±SD of mCherry+/GFP + cells. Significance was determined *via* unpaired two-tailed *t*-test and Gaussian distribution: ***p < 0.001. Each data point in the bar graphs was calculated from an individual retina (*n* = 2). ONL, outer nuclear layer; INL, inner nuclear layer; GCL, ganglion cell layer Scale bar = 40 μm.

To further confirm that this observed ectopic mCherry expression was driven by the GFAP minipromoter, we retested several constructs that showed leaky neuronal expression using Cre-dependent flexed AAV vectors (Schnütgen et al., 2003). Flexed AAV constructs rely on Cre-mediated insert inversion to allow stable expression of an insert of interest under the control of a ubiquitous promoter, and in this case should restrict expression to Cre-expressing Müller glia following 4-OHT administration. To accomplish this, we intravitreally injected 2 month old *GlastCreERT2;Sun1-GFP* mice with 7m8 AAV2 Cre-dependent flexed vectors expressing either mCherry alone, or mCherry in combination with *Atoh7* and *Brn3b*, under the control of the ubiquitous EF1a promoter (Figures 5A,B). Beginning four days following injection, we conducted four daily consecutive injections of 4-OHT to induce expression of both the flexed AAV construct and the transgenic Sun1-GFP construct. At 21 days following the fourth dose of 4-OHT injection, we then examined expression of both mCherry and Sun1-GFP, and observed selective Müller glia-specific expression of mCherry in both constructs tested indicated by extensive colocalization of GFP and mCherry expression (Figures 5C,D). The *GlastCreERT2;Sun1-GFP* mice show selective and robust perinuclear GFP labeling which colocalizes with selective Müller glial marker Sox9 (Supplementary Figure S4A,B). Immunostaining for Brn3b showed colocalization with mCherry-positive cells in FLEX-Atoh7-Brn3b-mCherry-transduced cells (Supplementary Figure S5).

None of the constructs tested induced detectable levels of expression of the neuronal markers RBPMS, TFAP2A, or OTX2, nor was radial displacement of Sun1-GFP positive Müller glia nuclei consistent with proliferation or migration observed in any condition tested (Figures 2–4, data not shown). This indicated that none of the constructs tested induced detectable levels of Müller glia-to-neuron conversion in uninjured retinas. In direct contrast to previous reports (Xiao et al., 2021), we observe no evidence for transdifferentiation of Sun1-GFP-positive Müller glia to retinal ganglion cells following misexpression of *Atoh7* and *Brn3b*, expressed using either the GFAP minipromoter or the flexed EF1a-driven construct. Raw numbers for all cell counts in the study are included in Supplementary Table S1.

## Discussion

In this study, we observed insert-dependent ectopic neuronal expression of GFAP minipromoter-driven mCherry expression for different AAV constructs. While GFAP-mCherry, the standard control used for experiments of this sort, consistently showed robust and selective glial-specific expression, the great majority of mCherry expression was restricted to retinal amacrine and ganglion cells for 5 out of the 7 experimental overexpression constructs that were tested. This did not reflect Müller glia-to-neuron conversion, as these cells did not express Müller glia-derived Sun1-GFP, but instead implies that the insert sequences themselves dramatically altered the specificity of the GFAP minipromoter. This could reflect the action of cis-regulatory elements that bind transcription factors that promote neuronal-specific expression, the activity of the vector-encoded transcription factors on the GFAP minipromoter, or some combination of the two. It is unclear why this was not also observed to nearly the same extent following *Nrl* or *Oct4* overexpression. Similar conclusions have been drawn following re-examination of claims of astrocyte-to-neuron conversion in brain (Wang L.-L. et al., 2021; Chen et al., 2021; Hoang et al., 2021), and these findings raise serious concerns about the accuracy of previously reported claims of AAV-mediated Müller glia-to-neuron conversion (Yao et al., 2018; Fu et al., 2020; Zhou et al., 2020; Xiao et al., 2021).

Since AAV vectors currently represent the least toxic and most effective method of gene delivery *in vivo*, this poses important challenges for future research into cell-based therapies. While several studies have reported Müller glia-specific AAV-based minipromoter constructs derived from genes other than *GFAP* (Korecki et al., 2021; Lin et al., 2022), these too may show ectopic expression when used to overexpress specific genes. In mice, where transgenic reagents such as the *GlastCreERT2;Sun1-GFP* that can reliably and irreversibly label glia are readily available, prospective genetic lineage analysis should be performed routinely in conjunction with AAV-based manipulations. In this case, Cre-dependent FLEX-AAV constructs can also be used to further restrict the action of AAV-based constructs to glial cells and avoid ectopic neuronal expression. In species where genetic models are not readily available, or in preclinical studies in humans, this requires other approaches. These could include detailed morphological, physiological, and/or single cell RNA-Seq-based identification of reprogrammed glia and immature neurons.

## Materials and methods

### Mice

Mice were raised and housed in a climate-controlled pathogen-free facility on a 14/10 h light/dark cycle. Mice used in this study were *GlastCreERT2;Sun1-GFP*, which were generated by crossing the *GlastCreERT2* and *Sun1-GFP* lines developed by Dr. Jeremy Nathans at Johns Hopkins (de Melo et al., 2012; Mo et al., 2015), and were obtained from his group. Maintenance and experimental procedures performed on mice were in accordance with the protocol approved by the Institutional Animal Care and USe Committee (IACUC) at the Johns Hopkins School of Medicine.

### GFAP-AAV delivery

#### Intraperitoneal 4-hydroxytamoxifen injection

To induce *Cre* recombination, *GlastCreERT2;Sun1-GFP* mice at ∼3 weeks of age were intraperitoneally injected with 4-OHT (Sigma-Aldrich, #H6278-50mg) in corn oil (Sigma-Aldrich, #C8267-500ML) at 1 mg/dose for three consecutive days.

#### Cloning, production and intravitreal injection of adeno-associated virus

The Addgene #50473 construct which contains a GFAP promoter was used in this study. The EGFP sequence was replaced by the mCherry sequence. The P2A and T2A ribosomal self-cleaving peptides are used to simultaneously express the transcription factor(s) 5′ to the mCherry reporter as a single polypeptide, which is then cleaved to generate the transcription factor and mCherry. Coding sequences of different transcription factors were synthesized by GeneWiz. AAV constructs were packaged into AAV2.7m8 by Boston Children’s Hospital Viral Core. Following 4-OHT i.p induction, two months old *GlastCreERT2;Sun1-GFP* mice were intravitreally injected with GFAP AAV constructs using a microsyringe with a 33G blunt-ended needle. Titre and injection volume for each construct are listed below:

**Table:** 

AAV-GFAP constructs	Titre (gc/ml)	Injection volume (μL)
GFAP-mCherry	1.96E^13	1μL/eye
GFAP-Oct4-P2A-mCherry	2.58E^13	1μL/eye
GFAP-Nrl-P2A-mCherry	2.58E^13	1μL/eye
GFAP-NeuroD1-P2A-mCherry	2.83E^13	1μL/eye
GFAP-Mybl1-P2A-mCherry	2.73E^13	1μL/eye
GFAP-Insm1-P2A-mCherry	5.76E^12	1μL/eye
GFAP-Atoh7-T2A-Ascl1-P2A-mCherry	7.60E^12	2μL/eye
GFAP-Atoh7-T2A-Brn3b-P2A-mCherry	8.50E^12	2μL/eye

#### FLEX-AAV delivery

Two months old *GlastCreERT2;Sun1-GFP* mice were intravitreally injected with AAV-FLEX constructs using a microsyringe with a 33G blunt-ended needle. Four days following AAV transduction, 4 once-daily intraperitoneal injections of 4-OHT (1mg/dose in corn oil) were administered to activate Cre recombinase. Retinas were collected 21 days following the fourth dose for analysis. Titre and injection volume for each construct are listed below:

**Table:** 

AAV-GFAP constructs	Titre (gc/ml)	Injection volume (μL)
EF1a-DIO-mCherry (Addgene#50462)	1.30E^13	1μL/eye
EF1a-DIO-Atoh7-T2A-Brn3b-P2A-mCherry	6.70E^12	2μL/eye

#### Fixation, sectioning, immunohistochemistry and imaging

Collection and immunohistochemical analysis of retinas was performed as described previously (Hoang et al., 2020). Briefly, mouse eye globes were collected at 8 days (2 mice per construct, *n* = 4 eyes) and 21 days (3 mice per construct, *n* = 6 eyes) post AAV infection and were fixed in 4% paraformaldehyde for 4 h at 4°C. Retinas were dissected in 1x PBS and incubated in 30% sucrose overnight at 4°C. Retinas were then embedded in OCT (VWR, #95057-838), cryosectioned at 16 μm thickness, and stored at −20°C. Sections were dried for 30 min in a 37°C incubator and washed 3 × 5 min with 0.1% TritonX-100 in PBS (PBST) and incubated in 10% Horse Serum (ThermoFisher, #26050070), 0.4% TritonX-100 in 1x PBS (blocking buffer) for 2 h at room temperature (RT). Sections were then incubated with primary antibodies in the blocking buffer overnight at 4°C. Primary antibodies used were Chicken anti-GFP (ThermoFisher, #A10262, 1:400), Rabbit anti-RFP (Abcam, #ab124754, 1:400), Goat anti-RFP (Rockland, #200-101-379, 1:400), Goat anti-Otx2 (R&D Systems, #AF 1979, 1:200), Rabbit anti-Rbpms (ThermoFisher, #15187-1-AP, 1:400), Mouse anti-Tfap2a (Abnova, #H00007020-M01, 1:200), Rabbit anti-Sox9 (Sigma-Aldrich, #AB5535, 1:400), Goat anti-Nrl (R&D System, #AF2945, 1:400), Goat anti-Brn3b (Santa Cruz, #sc-31989, 1:200).

Excess antibodies were removed by washing sections 4 × 5 min with PBST before secondary antibodies incubation in the blocking buffer for 2 h at RT. Secondary antibodies used were Donkey anti-Chicken 488 (Sigma-Aldrich, #SAB4600031-250UL, 1:400), Donkey anti-Rabbit 568 (ThermoFisher, #A-10042, 1:400), Donkey anti-Goat 568 (ThermoFisher, #A11057, 1:400), Donkey anti-Goat 633 (ThermoFisher, A-21082, 1:400), Donkey anti-Rabbit 647 (ThermoFisher, #A-31573, 1:400), Donkey anti-Mouse 647 (ThermoFisher, #A-31571, 1:400). Sections were then counterstained with DAPI in PBST, washed 4 × 5 min in PBST and mounted with ProLong Gold Antifade Mountant with DAPI (Invitrogen, #P36935) under coverslips, air-dried, and stored at 4°C. Fluorescent images were captured using a Zeiss LSM 700 confocal microscope.

#### Cell quantification and statistical analysis

Sox9+/GFP+, Tfap2a+/mCherry+, Rbpms+/mCherry cells were counted and divided by the total number of mCherry + cells from a single random, fixed size section per retina. mCherry+/GFP + cells were counted and divided by the total number of GFP+ (transduction efficiency) and mCherry + cells (transduction specificity) from three random sections per retina. Each data point in the bar graphs was calculated from an individual retina. All cell quantification data were graphed and analyzed using GraphPad Prism 9. Analysis between 2 samples used unpaired two-tailed *t*-test and Gaussian distribution. One-way ANOVA with Dunnett’s test were used for analysis between 3 or more samples. Two-way ANOVA were used for analysis between 3 or more samples of multiple groups. All results are presented as mean ± SD. Raw numbers for all cell counts in the study are included in Supplementary Table S1.

## Data Availability

The original contributions presented in the study are included in the article/Supplementary Material, further inquiries can be directed to the corresponding author.
