# Supplementary material for: Ectopic insert-dependent neuronal expression of GFAP promoter-driven AAV constructs in adult mouse retina
Source: Front Cell Dev Biol. 2022 Sep 19;10:914386. doi: 10.3389/fcell.2022.914386 (PMC9527291; doi:10.3389/fcell.2022.914386)
Supplement: Supplementary file 1 [file DataSheet1.PDF]

Supplemental figure legends:

Figure S1: Selective and robust perinuclear GFP labeling and colocalization with Müller glial marker Sox9 following GFAP AAV infection.

(A) Representative immunostaining for GFP and Sox9 expression in retinas following GFAP AAV infection. **(B)** Quantification of the number GFP+ cells co-labeled with Sox9+ cells showed robust and selective expression in Sun1-GFP and Sox9-positive Müller glia. White arrowheads indicate GFP+ & Sox9+ cells. **(C)** Representative whole cross section immunostained for mCherry expression following GFAP AAV infection. Some Sun1-GFP-positive astrocyte nuclei are observed in the GCL. INL, inner nuclear layer; GCL, ganglion cell layer. Scale bar = 40µm (A), 500µm (C).

Figure S2: Ectopic mCherry expression from GFAP promoter constructs expressing Insm1 in amacrine and retinal ganglion cells is evident at 8 days following AAV transduction.

Representative immunostaining for GFP, mCherry and 3 neuronal markers: **(A)** Tfap2a, **(B)** Rbpms, and **(C)** Otx2 expression in the retinas collected 8 days post GFAP AAV infection. GFAP-mCherry showed almost complete colocalization of construct-derived mCherry and Müller glia-specific Sun1-GFP, while GFAP-Insm1-mCherry showed little to no colocalization. Robust mCherry expression is observed in amacrine cells and at much greater levels of expression in ganglion cells with these GFAP AAV constructs. White arrowheads indicate co-labeled mCherry+ & GFP+ cells. Yellow arrowheads indicate co-labeled mCherry+ & marker+ cells. Quantification of transduction specificity (mCherry+ & GFP+ cells/ mCherry+ cells) **(D)**. Quantification of mean percentage ± SD of Tfap2a+/mCherry+, Rbpms+/mCherry+ and Otx2+/mCherry+ cells **(E)**. Significance was determined via unpaired two-tailed t-test and Gaussian distribution or two-way ANOVA with Dunnett's test: *$p < 0.05$, ****$p < 0.0001$. Each data point in the bar graphs was calculated from an individual retina (n = 4). INL, inner nuclear layer; GCL, ganglion cell layer. Scale bar = 40µm.

Figure S3: Ectopic mCherry expression from GFAP promoter constructs tandemly expressing Atoh7-Ascl1 and Atoh7-Brn3b in amacrine and retinal ganglion cells is evident at 8 days following AAV transduction.

Representative immunostaining for GFP, mCherry and 3 neuronal markers: **(A)** Tfap2a, **(B)** Rbpms, and **(C)** Otx2 expression in the retinas collected 8 days post GFAP AAV infection. GFAP-Atoh7-Ascl1-mCherry and GFAP-Atoh7-Brn3b-mCherry showed little to no colocalization of construct-derived mCherry and Müller glia-specific Sun1-GFP. mCherry expression is observed in amacrine cells and at higher levels in ganglion cells with these GFAP AAV constructs. Yellow arrowheads indicate co-labeled mCherry+ & marker+ cells. Quantification of transduction specificity (mCherry+ & GFP+ cells/ mCherry+ cells) **(D)**. Quantification of mean percentage ± SD of Tfap2a+/mCherry+, Rbpms+/mCherry+ and Otx2+/mCherry+ cells **(E)**. Significance was determined via unpaired two-tailed t-test and Gaussian distribution or two-way ANOVA with Dunnett's test: **$p < 0.01$, ****$p < 0.0001$. Each data point in the bar graphs was calculated from an individual retina (n = 4). INL, inner nuclear layer; GCL, ganglion cell layer. Scale bar = 40µm.

Figure S4: Selective and robust perinuclear GFP labeling and colocalization with Müller glial marker Sox9 in FLEX AAV construct.
(A) Representative immunostaining for GFP and Sox9 expression in retinas following infection with FLEX AAV construct. **(B)** Quantification of the number GFP+ cells co-labeled with Sox9+ cells showed robust and selective expression in Sun1-GFP and Sox9-positive Müller glia. White arrowheads indicate GFP+ & Sox9+ cells. **(C)** Representative whole cross section immunostained for mCherry expression following GFAP AAV infection. INL, inner nuclear layer; GCL, ganglion cell layer. Scale bar = 40μm (A), 500μm (C).

Figure S5: Immunostaining detects expression of Nrl and Brn3b in AAV-transduced Müller glia.
Representative immunostaining for GFP, mCherry and 2 transcription factors: Nrl and Brn3b expression in the retinas infected with GFAP-Nrl-mCherry and FLEX-Atoh7-Brn3b-mCherry constructs. White arrowheads indicate GFP+/mCherry+/TF insert+ triple positive cells. TF, transcription factor; INL, inner nuclear layer; GCL, ganglion cell layer. Scale bar = 40μm.

Table S1: Cell counts for data shown in the study.
Individual cell count number for calculation of transduction efficiency, specificity and colocalization of each cell type-specific marker. D8, 8 days and D21, 21 days.

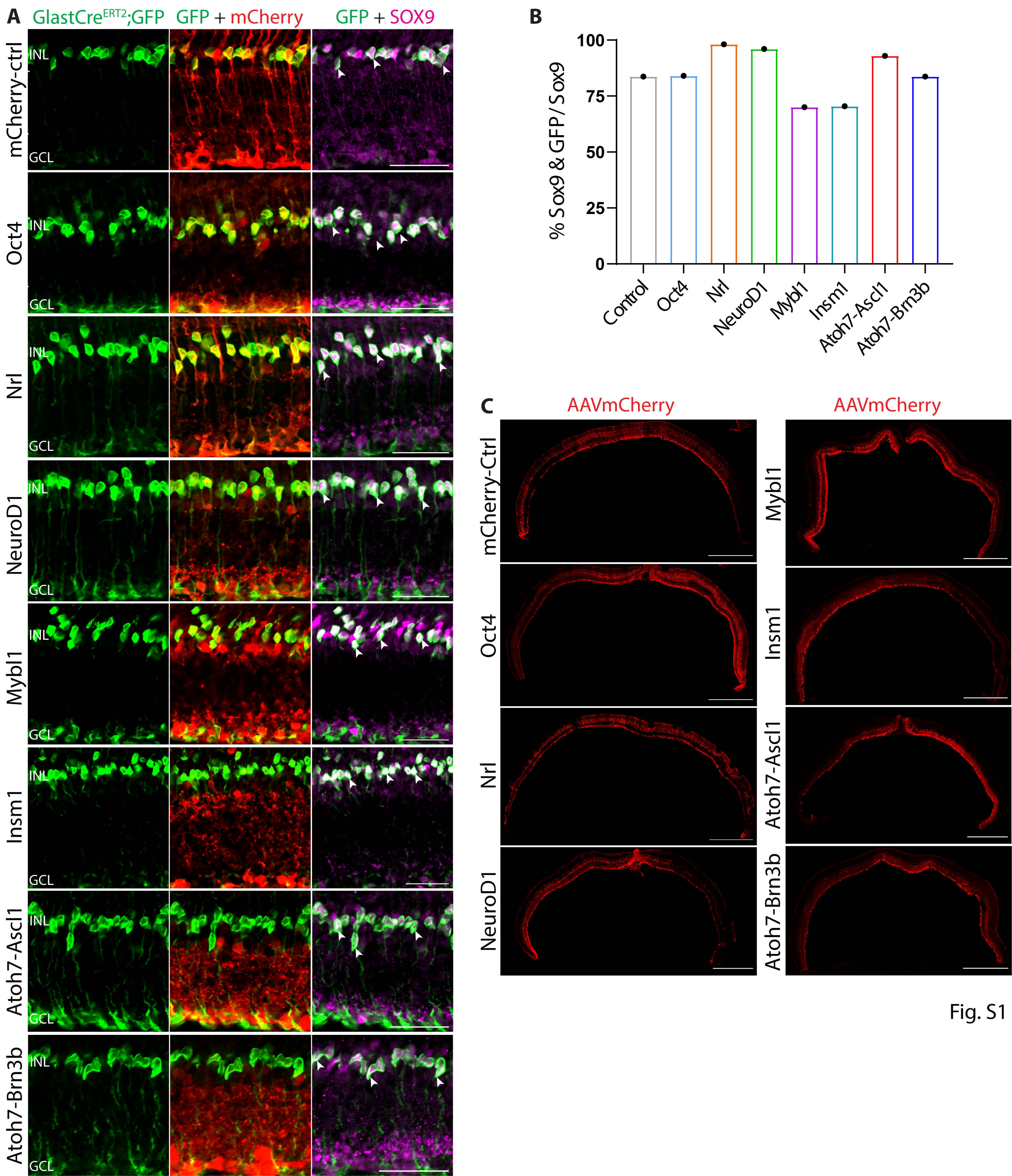
A
GlastCre^{ERT2};GFP
GFP + mCherry
GFP + SOX9
mCherry-ctrl
Oct4
Nrl
NeuroD1
Mybl1
Insm1
Atoh7-Ascl1
Atoh7-Brn3b
INL
GCL
B
% Sox9 & GFP/ Sox9
100
75
50
25
0
Control
Oct4
Nrl
NeuroD1
Mybl1
Insm1
Atoh7-Ascl1
Atoh7-Brn3b
C
AAVmCherry
AAVmCherry
mCherry-Ctrl
Oct4
Nrl
NeuroD1
Mybl1
Insm1
Atoh7-Ascl1
Atoh7-Brn3b

Fig. S1

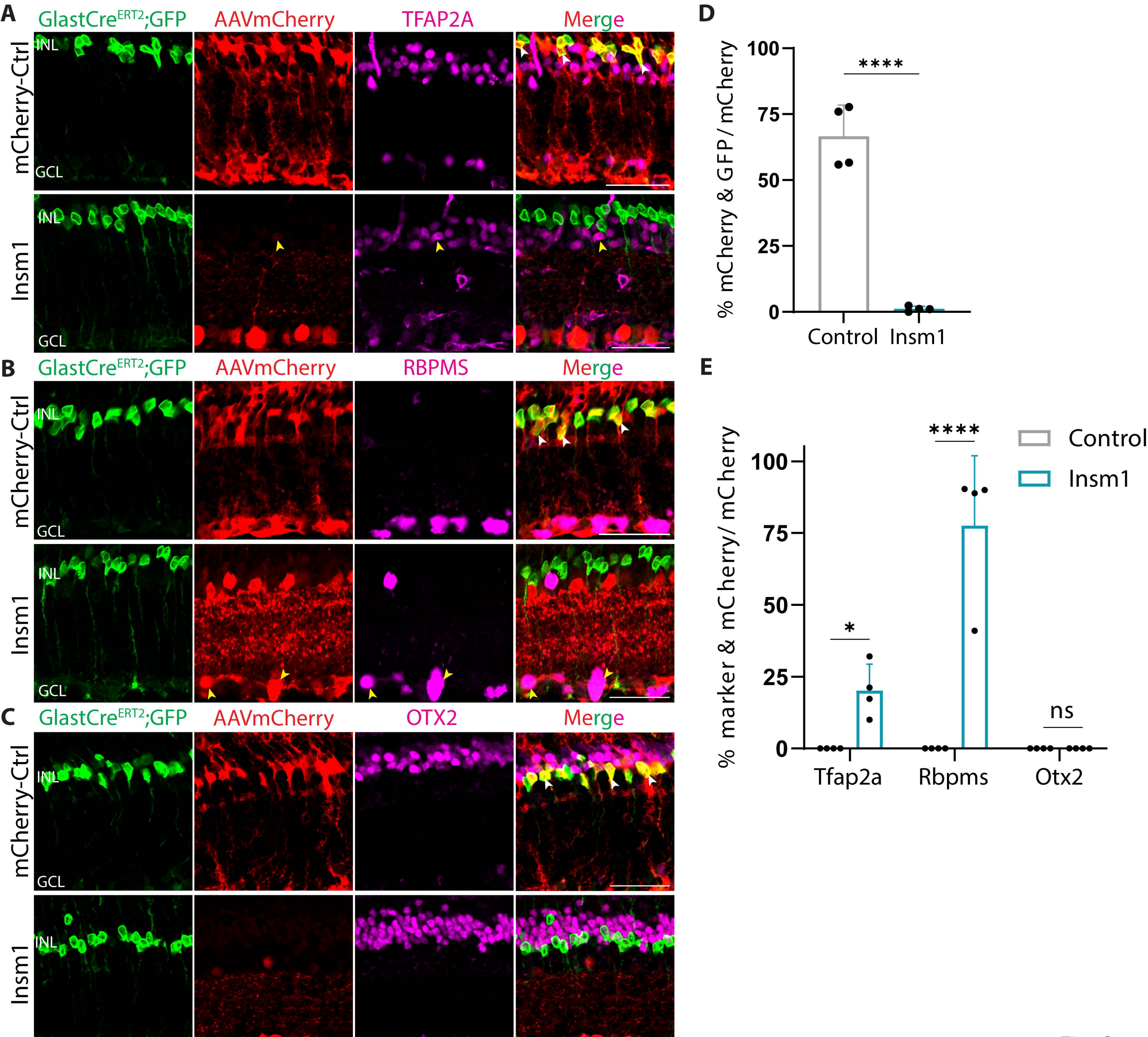
A
GlastCreERT2;GFP
AAVmCherry
TFAP2A
Merge
mCherry-Ctrl
Insm1
INL
GCL
B
GlastCreERT2;GFP
AAVmCherry
RBPMS
Merge
C
GlastCreERT2;GFP
AAVmCherry
OTX2
Merge
D
% mCherry & GFP / mCherry
100
75
50
25
0
****
Control
Insm1
E
% marker & mCherry/ mCherry
Control
Insm1
*
****
ns
Tfap2a
Rbpms
Otx2

Fig. S2

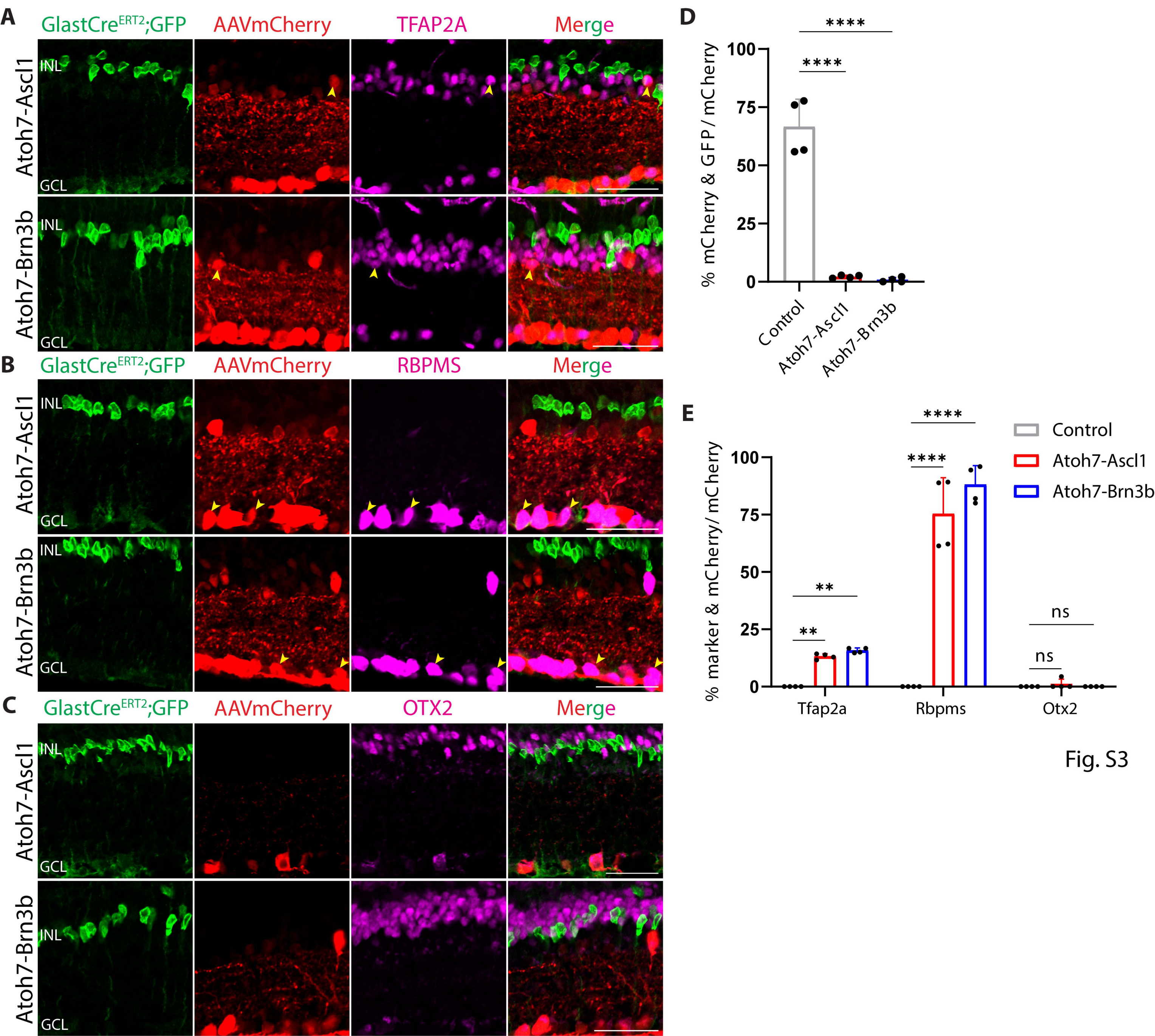

A
GlastCre^ERT2;GFP
AAVmCherry
TFAP2A
Merge
Atoh7-Ascl1
Atoh7-Brn3b
INL
GCL
B
GlastCre^ERT2;GFP
AAVmCherry
RBPMS
Merge
Atoh7-Ascl1
Atoh7-Brn3b
INL
GCL
C
GlastCre^ERT2;GFP
AAVmCherry
OTX2
Merge
Atoh7-Ascl1
Atoh7-Brn3b
INL
GCL
D
% mCherry & GFP / mCherry
100
75
50
25
0
****
****
Control
Atoh7-Ascl1
Atoh7-Brn3b
E
% marker & mCherry/ mCherry
100
75
50
25
0
**
**
****
****
ns
ns
Tfap2a
Rbpms
Otx2
Control
Atoh7-Ascl1
Atoh7-Brn3b

Fig. S3

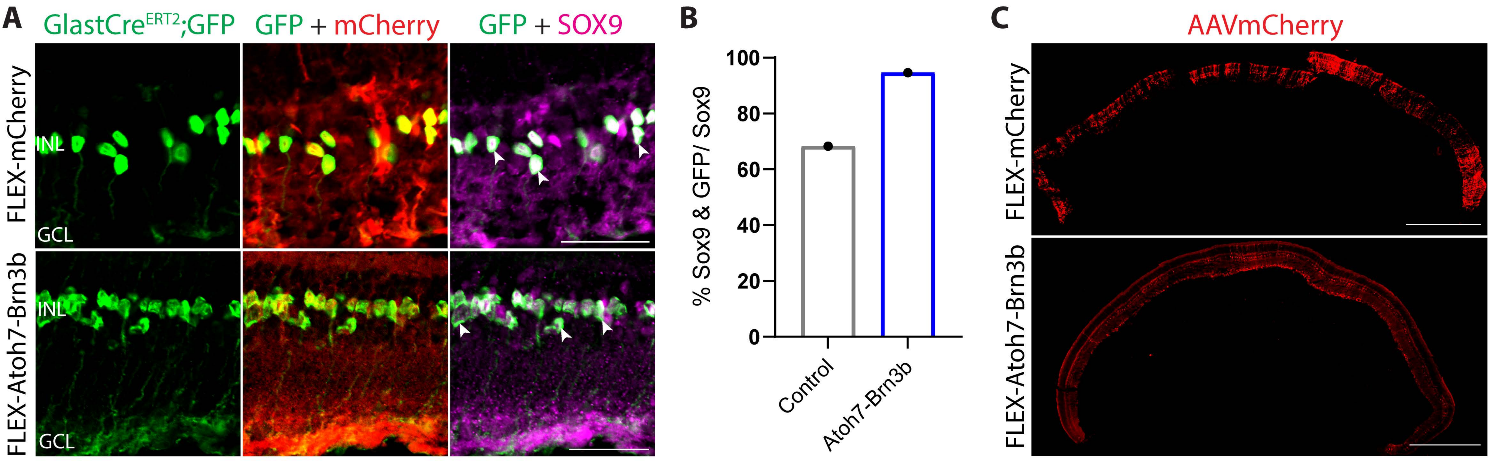
A
GlastCre^ERT2;GFP
GFP + mCherry
GFP + SOX9
FLEX-mCherry
FLEX-Atoh7-Brn3b
INL
GCL
B
% Sox9 & GFP/ Sox9
100
80
60
40
20
0
Control
Atoh7-Brn3b
C
AAVmCherry
FLEX-mCherry
FLEX-Atoh7-Brn3b

Fig. S4

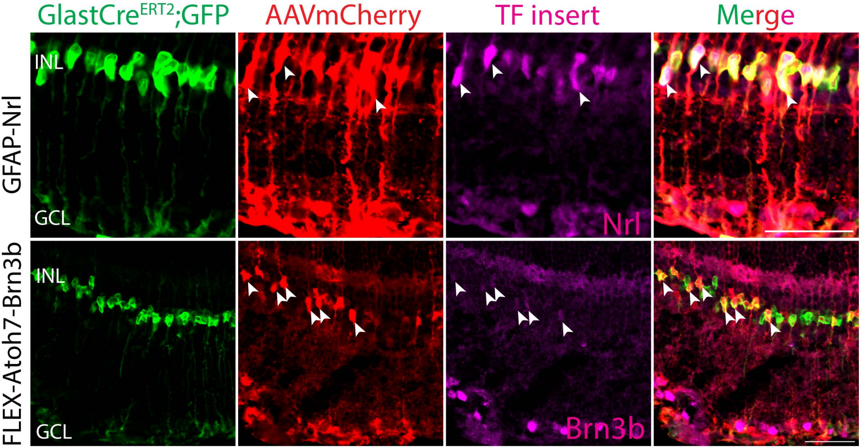
GlastCreERT2;GFP
AAVmCherry
TF insert
Merge
GFAP-Nrl
FLEX-Atoh7-Brn3b
INL
GCL
INL
GCL
Nrl
Brn3b

Fig. S5
